# KMU-191 Induces Apoptosis in Human Clear Cell Renal Cell Carcinoma Caki Cells Through Modulation of Bcl-xL, Mcl-1 (L), c-FLIP (L), and p53 Proteins

**DOI:** 10.7150/jca.85650

**Published:** 2023-07-16

**Authors:** Shin Kim, Dong Eun Kim, Hyunsu Kang, Victor Sukbong Hong, Jieun Jeon, Jinho Lee, Ki-Suk Kim, Jong-Wook Park

**Affiliations:** 1 Department of Immunology, School of Medicine, Keimyung University, 1095 Dalgubeol-daero, Daegu 42601, Republic of Korea.; 2 Institute of medical science, Keimyung University, School of Medicine, Keimyung University, 1095 Dalgubeol-daero, Daegu 42601, Republic of Korea.; 3 Institute for Cancer Research, Keimyung University, School of Medicine, Keimyung University, 1095 Dalgubeol-daero, Daegu 42601, Republic of Korea,; 4 R&D Center for Advanced Pharmaceuticals & Evaluation, Korea Institute of Toxicology, Daejeon 34114, Korea.; 5 Department of Physiology, Sungkyunkwan University School of Medicine, Suwon 16419, Korea.; 6 Department of Chemistry, Keimyung University, 1095 Dalgubeol-daero, Daegu 42601, Republic of Korea.

**Keywords:** KMU-191, Bcl-xL, Mcl-1 (L), c-FLIP (L), p53, Apoptosis, Renal cancer

## Abstract

The anti-proliferative effects of a newly developed *N*3-acyl-*N*5-aryl-3,5-diaminoindazole analog, KMU-191, have been previously evaluated in various cancer cells. However, the detailed anti-cancer molecular mechanisms of KMU-191 remain unknown. In this study, we investigated anti-cancer mechanisms by which KMU-191 regulates apoptosis-related genes in human clear cell renal cell carcinoma Caki cells. KMU-191 induced poly ADP-ribose polymerase cleavage and caspase-dependent apoptosis. In addition, KMU-191 induced down-regulation of the long form of cellular FADD-like IL-1β-converting enzyme inhibitory protein (c-FLIP (L)) at the transcriptional level as well as that of long form of myeloid cell leukemia (Mcl-1 (L)) and B-cell lymphoma-extra large at the post-transcriptional level. Furthermore, KMU-191-induced apoptosis was closely associated with the Mcl-1 (L) down-regulation, but also partially associated with c-FLIP (L) down-regulation. In contrast, KMU-191 up-regulated p53, which is closely related to KMU-191-induced apoptosis. Although KMU-191 showed cytotoxicity of normal cells, it unusually did not induce cardiotoxicity. Taken together, these results suggest that a multi-target small molecule, *N*3-acyl-*N*5-aryl-3,5-diaminoindazole analog, KMU-191 is a potential anti-cancer agent that does not induce cardiotoxicity.

## Introduction

The discovery of aminoindazole-based anti-cancer drugs that target several kinases and elucidation of their anti-cancer mechanisms are widely pursued to establish a scientific basis for their anti-cancer property 1-4. Previous study has revealed that a novel compound, *N*3-acyl-*N*5-aryl--3,5-diaminoindazole analog (KMU-191) has anti-proliferative effects in various cancer cell lines 5. However, the anti-cancer mechanisms of KMU-191 have not been fully investigated. In this study, we demonstrated that anti-cancer mechanisms of KMU-191 are associated with apoptosis in human clear cell renal cell carcinoma cells.

Apoptosis, an important programmed cell death process, is involved in various biological processes, including homeostasis through normal cell turnover, adequate embryonic development, immune system function, and elimination of unwanted or damaged cells 6-9. Apoptosis occurs through two major pathways: the extrinsic pathway, in which components such as FLIP and caspase-8 are involved, and the intrinsic pathway in which the bcl-2 family is involved, such as B cell lymphoma-2 (Bcl-2), B-cell lymphoma-extra large (Bcl-xL), and myeloid cell leukemia 1 (Mcl-1) 10, 11. In addition, the tumor suppressor gene p53 plays an important role in apoptosis in response to DNA damage 12.

Unexpected cardiotoxicity is a critical cause of the termination of clinical trials and drug withdrawal from the market 13. Torsade de pointes (TdP), a specific form of polymorphic ventricular tachycardia, prolongs the QT interval by inhibiting ion channels associated with the action potential duration in the myocardial cell membrane and inducing cardiotoxicity associated with the side effects of chemicals 14. Predicting TdP incidence is important for assessing cardiotoxicity and safety in the development of new drugs. Therefore, using electrocardiography (ECG) and left ventricle pressure (LVP), it is crucial to ensure the electrophysiological safety of drugs based on a reliable assessment of their proarrhythmic cardiotoxicity potential 15, 16.

This study aimed to investigate the molecular mechanism underlying the anti-cancer effect of KMU-191 and its electrophysiological safety for the first time. The results revealed that KMU-191 induces apoptosis in human clear cell renal cell carcinoma Caki cells through the modulation of Mcl-1 (L), c-FLIP (L), and p53, and had no effect on cardiotoxicity.

## Materials and Methods

### Synthesis of KMU-191

**Figure 1:**
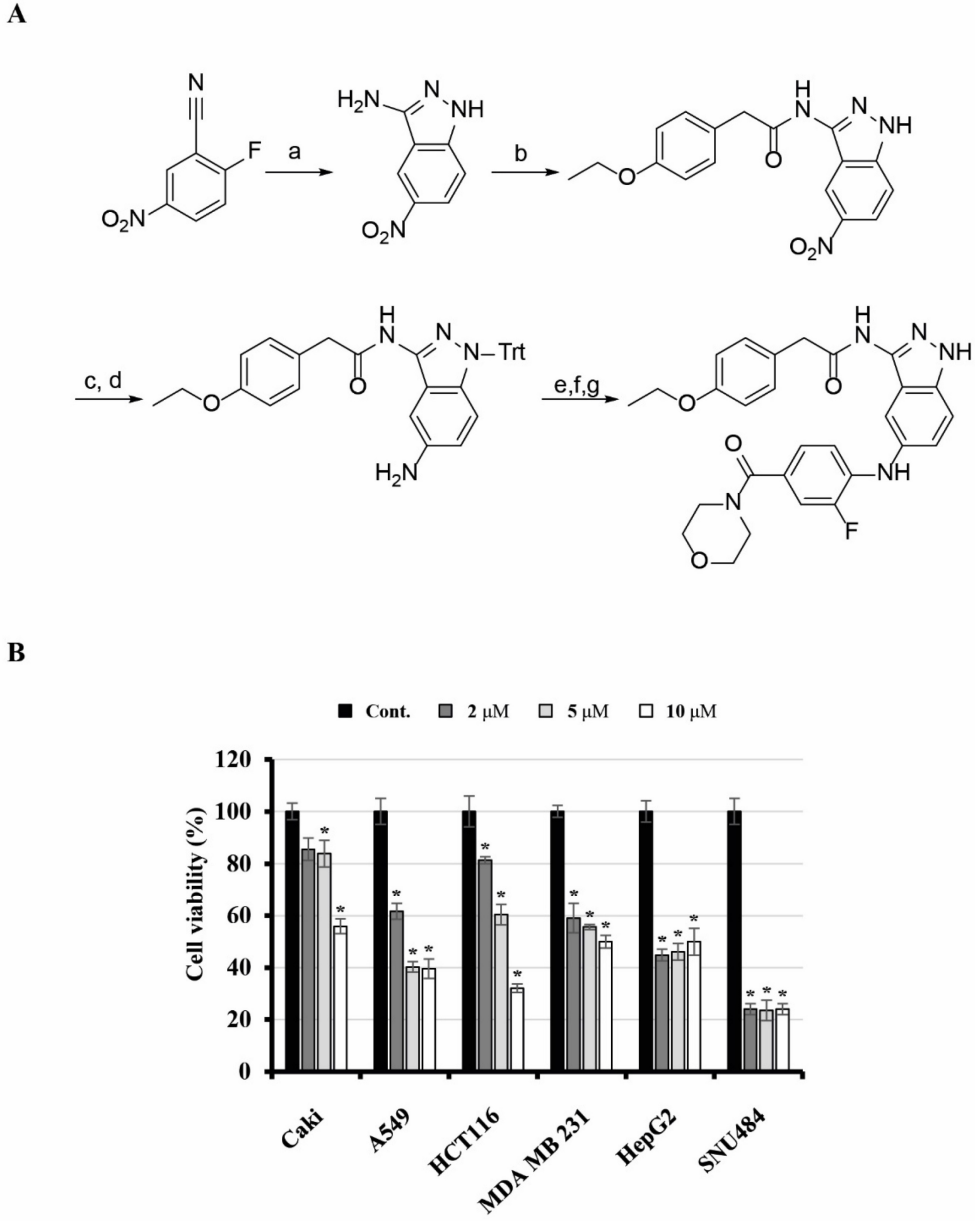
** Synthesis of KMU-191 and its anti-proliferative effect on various cancer cells.** (A) Synthetic scheme for KMU191. Reagents and experimental conditions: a) H_2_NNH_2_, *n*-BuOH, reflux, b) i) *p*-EtOC_6_H_4_CH_2_COCl, THF, reflux, ii) 2N NaOH, c) TritCl, AcCN, reflux, d) H_2_, Pd/C, MeOH, e) 4-Br-3-FC_6_H_3_CO_2_Et, Pd_2_(dba)_3_, (*R*)-BINAP, NaOBu-*t*, toluene, reflux, f) LiOH, THF/H_2_O/MeOH, g) i) morpholine, EDC, HOBt, DMF, ii) TFA, DCM. (B) Effect of KMU-191 on the growth of various cancer cells. Caki, A549, p53 wild-type HCT116 (HCT116), MDA MB 231, HepG2, and SNU484 cells were treated with the indicated concentrations of KMU-191 for 48 h. Cell viability was determined by SRB assay. Values in the graph (B) indicate the mean ± SD of three independent experiments. * P < 0.05 compared to the respective control. Cont., control; SRB, Sulforhodamine B.

2-(4-Ethoxyphenyl)-*N*-(5-((2-fluoro-4-(morpholine-4-carbonyl)phenyl)amino)-1*H*-indazol-3-yl)acetamide (KMU-191) was synthesized as shown in Figure 1. 3-Amino-5-nitroindazole was synthesized from 2-fluoro-5-nitrobenzonitrile and hydrazine. Monoacylation at the 3-amino position of the indazole was performed via consecutive diacylation and deacylation reactions. After protecting the free amino group of the indazole with triphenylmethyl (trityl) chloride, palladium-catalyzed reduction of the nitro group gave a 5-amino group that was arylated by a palladium-catalyzed Buchwald-Hartwig reaction 17. An amide coupling reaction after hydrolysis of the ester and deprotection of the trityl group produced KMU-191.

### Cell lines and culture

A549 human non-small cell lung cancer cells (p53 wild-type) and HCT116 human colorectal carcinoma cells (p53 wild-type and p53 deficient) obtained from the American Type Culture Collection (ATCC, Rockville, MD, USA) were grown in an RPMI 1640 medium (Welgene Inc., Gyeongsan, Korea) supplemented with 10% heat-inactivated fetal bovine serum (FBS; Welgene Inc., Gyeongsan, Korea), 2 mM L-glutamine, 100 µg/mL streptomycin and 100 µg/mL penicillin. Caki human renal clear cell carcinoma cells (p53 wild-type), MDA MB 231 human breast cancer cells (mutant p53) 18, and HepG2 human hepatocellular carcinoma cells (p53 wild-type) obtained from the ATCC (Rockville, MD, USA). The normal mouse kidney TCMK-1 cells were kindly donated from Dr. T. J. Lee (Yeungnam University, Korea), and human mesangial cells were purchased from Lonza (Basel, Switzerland). Cells were grown in Dulbecco's modified Eagle's medium (DMEM; Welgene Inc., Gyeongsan, Korea), containing 10% heat-inactivated FBS, 20 mM HEPES buffer, 100 µg/mL streptomycin, and 100 µg/mL penicillin. SNU484 human gastric adenocarcinoma cells (mutant p53) 19, provided by Korean Cell Line Bank (Seoul, Korea), were grown in RPMI 1640 medium (Welgene Inc., Gyeongsan, Korea) supplemented with 10% heat-inactivated FBS, 2 mM L-glutamine, 100 µg/mL streptomycin, and 100 µg/mL penicillin.

### Reagents

Anti-Bcl-xL (sc-634, 1:700), anti-p53 (sc-126, 1:1000), anti-p21 (sc-6246, 1:700), anti-Mcl-1 (sc-819, 1:1000), and anti-c-FLIP (sc-8347, 1:700) antibodies were purchased from Santa Cruz Biotechnology (Dallas, TX, USA). The anti-β-actin (A5441, 1:2000) antibody and glutathione ethyl ester (GEE) were purchased from Sigma Chemical Co. (St. Louis, MO, USA). The anti- poly (ADP-ribose) polymerase (PARP) (#9542, 1:1000) antibody was purchased from Cell Signaling Technology (Danvers, MA, USA). The anti-caspase-3 (610322, 1:1000) antibody was purchased from BD Biosciences (Bedford, MA, USA). Benzyloxycarbony-Val-Ala-Asp-fluoromethyl ketone (Z-VAD-FMK) was purchased from R&D Systems (Minneapolis, MN, USA). PD-98059 (a MEK inhibitor; PD), SP600125 (a JNK inhibitor; SP), and SB-203580 (a p38 MAP Kinase inhibitor; SB) were purchased from Enzo Life Sciences (Farmingdale, NY, USA). N-acetylcysteine (NAC) was purchased from Calbiochem (San Diego, CA, USA).

### Sulforhodamine B (SRB) assay

After the indicated incubation time, adherent cell cultures were fixed in situ by adding 50 µL of 50% (w/v) cold trichloroacetic acid (Sigma Chemical Co., St. Louis, MO, USA) and incubated for 60 min at 4 °C. The supernatant was discarded and the plates were washed five times with deionizedwater and dried. Fifty microliters of SRB (Sigma Chemical Co., St. Louis, MO, USA) solution (0.4%w/v) in 1% acetic acid (PanReac AppliChem, Barcelona, Spain) were added to each well and incubated for 30 min at room temperature. Plates containing SRB solution were washed five times with 1% acetic acid. Then, plates were air dried and 100 µL/well of 10 mM Tris base (pH 10.5) (Sigma Chemical Co., St. Louis, MO, USA) were added and the absorbance of each well was read on an enzyme-linked immunosorbent assay reader at 510 nm. Finally, cytotoxicity was measured as the percentage of absorbance compared to that of control cells.

### Western blotting analysis

Cellular lysates were obtained by suspending 0.5 × 10^6^ cells in 80 µL of RIPA buffer (20 mM HEPES and 0.5% Triton X-100, pH 7.6). The cells were disrupted by vortexing and extracted at 4 °C for 30 min. Protein concentrations were evaluated using the BCA assay kit (Thermo Fisher Scientific, Waltham, MA, USA). Proteins were electrotransferred onto Immobilon-P membranes (Millipore Corp., Bedford, MA, USA). Specific proteins were detected using an ECL Western blotting kit (EMD Millipore, Darmstadt, Germany) according to the manufacturer's instructions. The signal strength was analyzed using a Chemi Image System Fusion FX (Vilber Lourmat, Collégien, France).

### Flow cytometric analysis

Approximately 0.3 × 10^6^ cells were suspended in 100 µL Phosphate-buffered saline (PBS), and 200 µL of 95% ethanol was added while vortexing. The cells were incubated at 4 °C for 1 h, washed with PBS, and resuspended in 250 µL of 1.12% sodium citrate buffer (pH 8.4) along with 12.5 µg RNase. Incubation was continued at 37 °C for 30 min. Cellular DNA was then stained by adding 250 µL propidium iodide (50 µg/mL) for 30 min at room temperature. Stained cells were analyzed using a FACScan flow cytometer (Becton Dickinson and Co., Franklin Lakes, NY, USA) to determine the relative DNA content based on red fluorescence.

### RNA isolation and quantitative real-time PCR (qPCR)

Total cellular RNA was extracted from the tissues using TRIzol reagent (Molecular Research Center, Inc., Cincinnati, OH, USA). RNA was quantified using Nanodrop 1000 (Thermo Scientific, Wilmington, DE, USA). Each cDNA was synthesized from 2 µg of total RNA using M-MLV reverse transcriptase (Promega, Madison, WI, USA) according to the manufacturer's protocol. Table 1 lists the specific primer pairs and SYBR GREEN Premix used (TOYOBO, Japan). qPCR was performed on the LightCycler^®^ 480 real-time PCR system (Roche Diagnostics, Mannheim, Germany). β-actin was used as a housekeeping gene for normalization, and a no-template sample was used as a negative control. Then, the qPCR data were analyzed by the 2^-ΔΔct^ method 20.

### Establishment of stable cell lines overexpressing Mcl-1 (L) and c-FLIP (L)

The cDNA of Mcl-1 (L) was amplified by PCR using specific primers. The sequences of the sense and antisense primer for Mcl-1 (L) were 5'-GCGACTGGCAAAGCTTGGCCTAA-3' and 5'-CAACTCTAGAAACTGGTTTTGGTG-3', respectively. The PCR product was digested with Hind-III and XbaI, cloned into the pFLAG-CMV-4TM expression vector (Sigma Chemical Co., St. Louis, MO, USA), and termed pFLAG-CMV-4/Mcl-1 (L). Caki cells were stably transfected with the pFLAGCMV-4/Mcl-1 (L) plasmid using Lipofectamine per the manufacturer' instructions (ThermoFisher Scientific, Waltham, MA, USA). Human cDNA encoding c-FLIP (L) was PCR-amplified from plasmids (pCA-FLAGhFLIP (L), kindly provided by Dr. S. I. Park, Korea Centers for Disease Control and Prevention, Seoul, Korea) containing these sequences with specific primers. The c-FLIP (L) cDNA fragment was digested with KpnI and XhoI and subcloned into a pcDNA 3.1(+) vector (Invitrogen, Carlsbad, CA, USA). The resulting constructs were confirmed using nucleotide sequencing. Caki cells were stably transfected with the pCA-FLAGhFLIP (L) plasmid using Lipofectamine per the manufacturer's instructions (Invitrogen, Carlsbad, CA, USA). After 48 h of incubation, transfected cells were selected in cell culture medium containing 700 µg/mL G418 (Invitrogen, Carlsbad, CA, USA). After 2 or 3 weeks, single independent clones were randomly isolated and each clone was plated separately. After clonal expansion, cells from each independent clone were tested for the expression of Mcl-1 (L) and c-FLIP (L) proteins by immunoblotting and the cells were used for further experiments.

### Small interfering RNA (siRNA) Transfection

p53 siRNA duplexes were obtained from Cell Signaling Technology (Danvers, MA, USA). The control siRNA duplexes used in this study were purchased from Invitrogen (Carlsbad, CA, USA) and had the following sequence: green fluorescent protein, AAGACCCGCGCCGAGGUGAAG. The cells were transfected with siRNA oligonucleotides using Lipofectamine RNAiMAX (Invitrogen, Carlsbad, CA, USA) per the manufacturer's instructions.

### Langendorff heart preparation

Sprague Dawley rats of male (200 - 250 g) were injected intravenously with 1000 IU/kg heparin and then anesthetized by intravenous injection of 30 mg/kg sodium pentobarbital. After they were sedated and had lost pedal reflex activity, the hearts were rapidly excised and perfused via the aorta on a Langendorff apparatus (EMKA Technologies, Paris, France) using modified Krebs-Henseleit buffer saturated with carbogen (95% O_2_ and 5% CO_2_) containing 112 mM NaCl, 5 mM KCl, 11.5 mM glucose, 25 mM NaHCO_3_, 1.2 mM MgSO_4_, 1.2 mM KH_2_PO_4_, 2 mM pyruvic acid, and 1.25 mM CaCl_2_ at a constant perfusion pressure (60 - 80 mmHg). All the experiments were conducted after stabilization for 10 min.

### LVP and ECG recordings

To measure LVP, a water-filled latex balloon attached to a metal cannula was placed in the left ventricle through the pulmonary vein and connected to a pressure transducer (EMKA Technologies, Paris, France). ECGs were recorded using two surface electrodes (EMKA Technologies, Paris, France) held with a spring against the epicardium. One electrode was placed on the right ventricle near the atrium-ventricle ring and the second on the left ventricle in a position. All hemodynamic parameters were recorded and evaluated using iox2 software (EMKA Technologies, Paris, France) for 10 - 15 min before and after reperfusing KMU-191 with buffer. The QTc in milliseconds was calculated as follows: Bazett: QTcB = QT/RR^1/2^; Fridericia: QTcF = QT/RR^1/3^. KMU-191 was perfused serially from low concentration (1 μM) to high concentration (10 μM). The procedures used in this study were reviewed and approved by the Institutional Animal Care and Use Committee of the Korea Institute of Toxicology (RS21006).

### Statistical analysis

All data were statistically analyzed by one-way analysis of variance followed by post hoc comparisons (Student-Newman-Keuls test) using SPSS (ver. 27.0) software (SPSS, Inc., Chicago, IL, USA). Statistical significance was set at P-value < 0.05.

## Results

### Anti-cancer effect of KMU-191 in various human cancer cells

**Figure 2:**
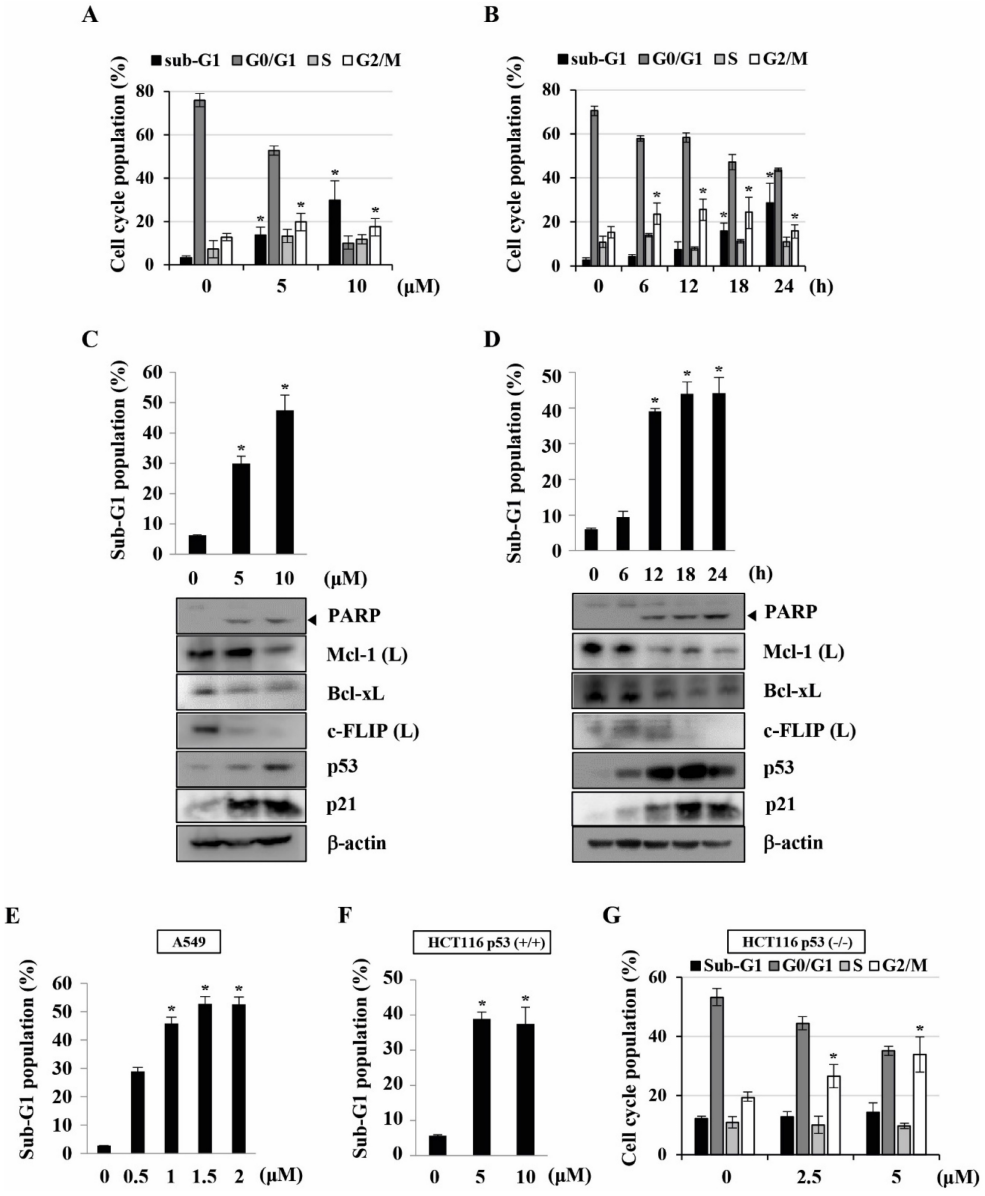
** KMU-191 induces apoptosis and regulates apoptosis-related proteins in cancer cells.** (A,C) Caki cells were treated with the indicated concentrations of KMU-191 for 24 h. (B,D) Caki cell were treated with 10 μM KMU-191 for the indicated time points. (E,F,G) A549, p53 wild-type HCT116, and p53 deficient HCT116 cells were treated with the indicated concentrations of KMU-191 for 24 h, respectively. Cell cycle populations was measured by flow cytometry (A,B,G). Sub-G1 populations and protein expression were measured by flow cytometry (C-F) and Western blotting analysis (C,D), respectively. Cleavage of PARP is indicated with an arrowhead. The expression level of β-actin was used as a control of protein loading. Values in the graph (A-G) indicate the mean ± SD of three independent experiments. * P < 0.05 compared to the respective control. HCT116 p53 (+/+), p53 wild-type HCT116 cells; HCT116 p53 (-/-), p53 deficient HCT116 cells.

Previously, we found that KMU-191 has an anti-proliferative effect on various human cancer cells 5. This study was designed to investigate the anti-proliferative effect of KMU-191 on various human cancer cell lines using the SRB assay. As shown in Figure 1B, KMU-191 inhibited the proliferation of Caki, A549, p53 wild-type HCT116, MDA MB 231, HepG2, and SNU484 cells in a dose-dependent manner. To determine whether KMU-191 induces apoptosis and cell cycle in cancer cells, flow cytometric analysis was performed. KMU-191 induced G2/M arrest of Caki cells in a dose- and time-dependent manner (Figure 2A and 2B). Moreover, KMU-191 induced G2/M arrest of p53 deficient HCT116 cells in a dose-dependent manner (Figure 2G). Additionally, KMU-191 induced apoptosis of Caki cells and PARP cleavage in a dose- and time-dependent manner (Figure 2C and 2D). Moreover, treating other cancer cells with KMU-191 resulted in a remarkable increase in the sub-G1 population (Figure 2E and 2F). Next, we investigated whether KMU-191 regulates apoptosis-related proteins in Caki cells. KMU-191 down-regulated the expression of Mcl-1 (L), Bcl-xL, and c-FLIP (L) in a dose- and time-dependent manner (Figure 2C and 2D). Caki cells were treated with KMU-191 resulted in up-regulation of p53 in a dose- and time-dependent manner (Figure 2C and 2D). Taken together, these results demonstrated that KMU-191 induces apoptosis and regulates the expression of apoptosis-related proteins.

### Modulation of Mcl-1 (L), Bcl-2, and c-FLIP (L) expression levels in KMU-191-treated Caki cells

**Figure 3:**
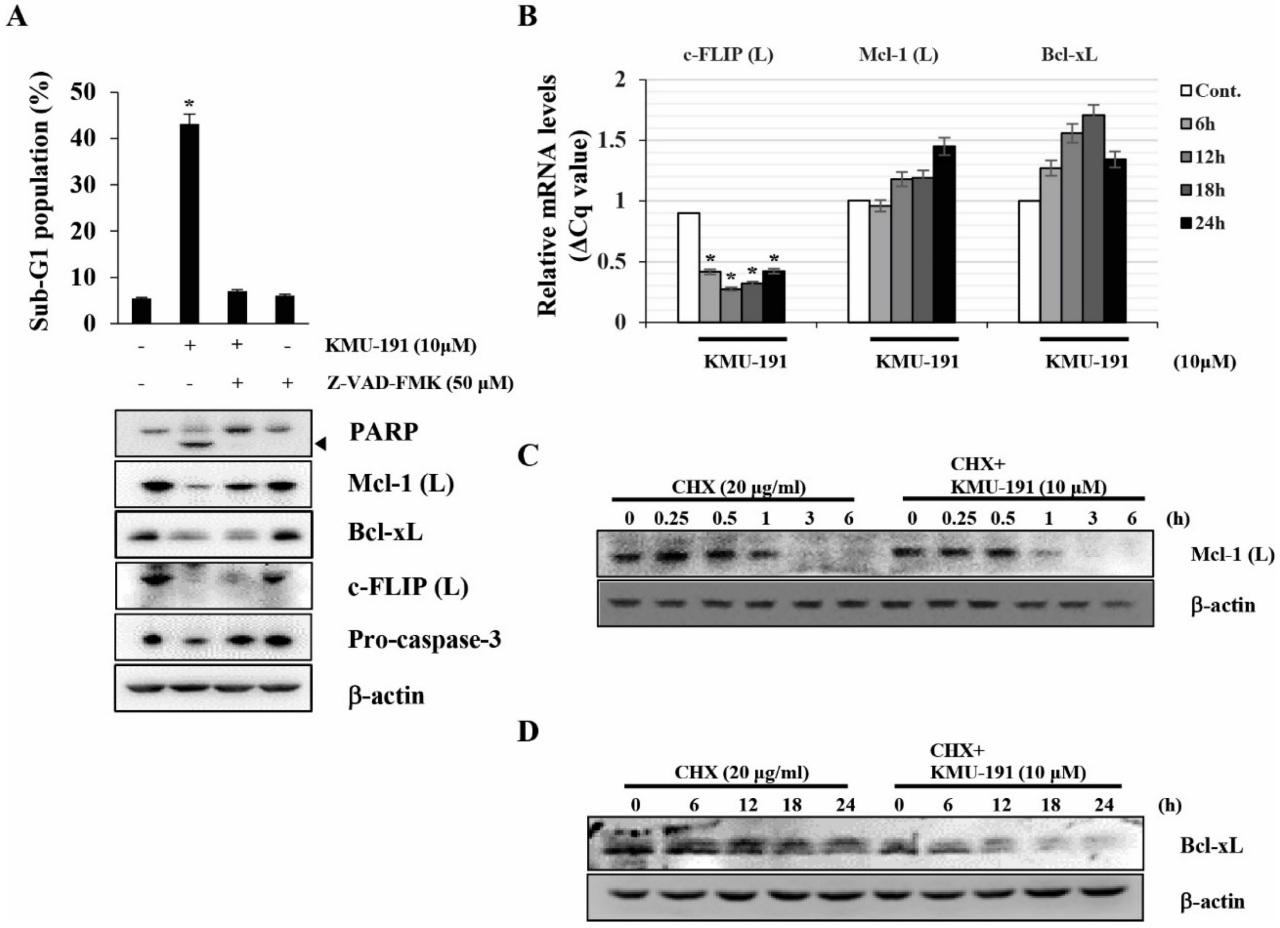
** KMU-191 regulates apoptosis-related proteins in Caki cells at the transcriptional and the post-transcriptional levels.** (A) Caki cells were incubated with Z-VAD-FMK or solvent for 30 min, then the cells were treated with KMU-191 for 24 h. (B) Caki cell were treated with 10 μM KMU-191 for the indicated time points. (C,D) Caki cells were treated with CHX in the presence or absence of KMU-191. Sub-G1 population and protein expression were measured by flow cytometry (A) and Western blotting analysis (C,D), respectively. mRNA levels were measured using quantitative real-time PCR (normalized to the corresponding β-actin mRNAs) (B). Cleavage of PARP is indicated with an arrowhead. The expression level of β-actin was used as a control of protein loading. Values in the graph (A,B) indicate the mean ± SD of three independent experiments. * P < 0.05 compared to the respective control. Cont., control; CHX, cyclohexamide.

Next, we elucidated the mechanisms underlying the regulation of apoptosis-related proteins modulated by KMU-191. Z-VAD-FMK inhibited KMU-191-induced apoptosis and down-regulation of Mcl-1 (L) as observed by FACS and western blot analysis, respectively (Figure 3A). Notably, the down-regulations of Bcl-xL and c-FLIP (L) were not suppressed by Z-VAD-FMK (Figure 3A). KMU-191 down-regulated c-FLIP (L) at the transcriptional level, while Mcl-1 (L) and Bcl-xL at post-transcriptional level (Figure 3C and 3D).

### The role of c-FLIP (L), Mcl-1 (L), and p53 in KMU-191-mediated apoptosis in Caki cells

**Figure 4:**
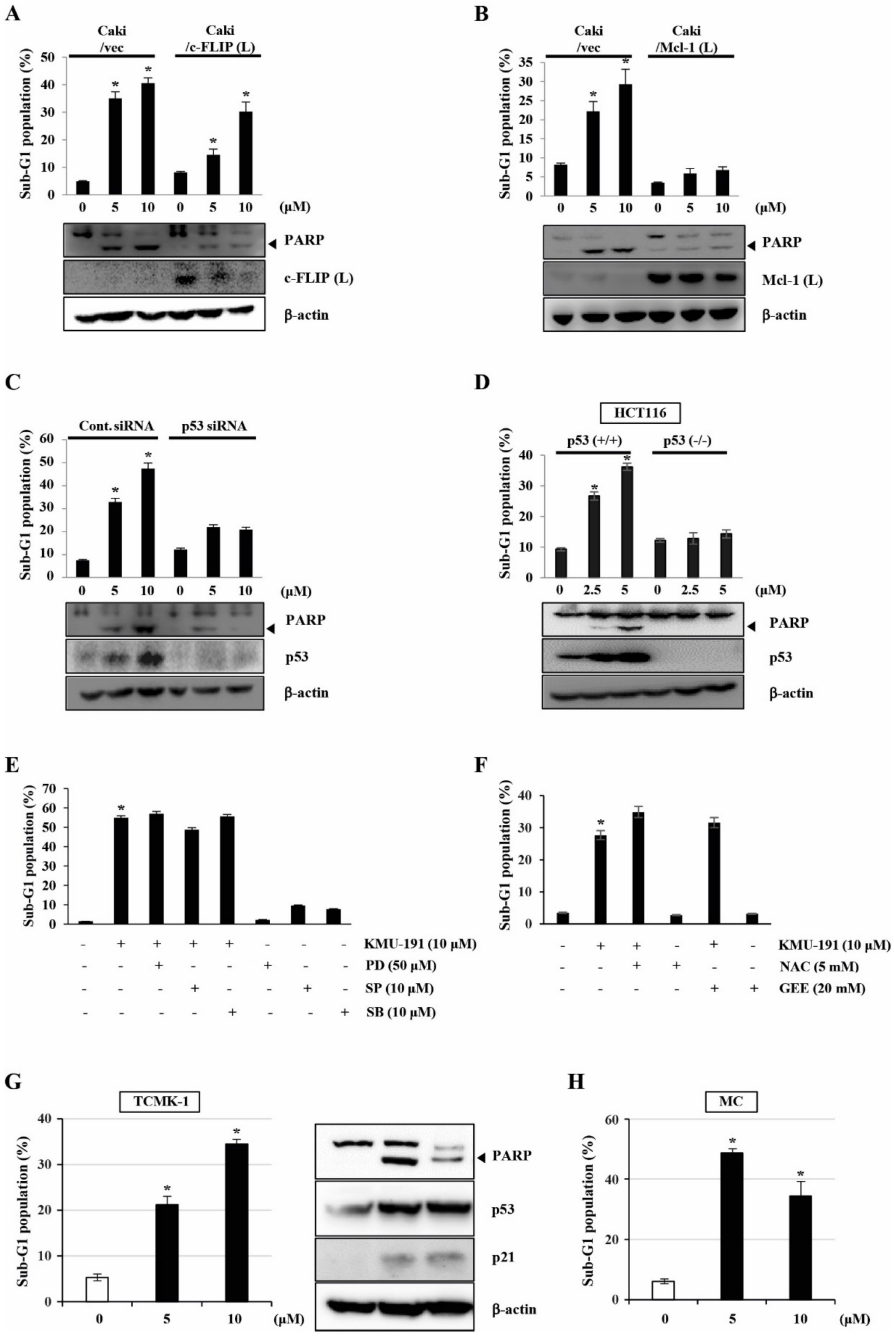
** Mcl-1 (L) and p53 play important roles in KMU-191-induced apoptosis of cancer cells.** (A,B) Vector cells (Caki/vec), c-FLIP (L)-overexpressing cells (Caki/c-FLIP (L)), and Mcl-1 (L)-overexpressing cells (Caki/Mcl-1 (L)) were treated with KMU-191 for 24 h. (C) After Caki cells were transfected with control (Cont. siRNA) or p53 siRNA for 18 h, the cells were then incubated for another 24 h (vehicle) or treated with the indicated concentrations of KMU-191 for 24 h. (D) p53 wild-type and p53 deficient HCT116 cells were treated with the indicated concentrations of KMU-191 for 24 h. (E) Caki cells were treated with the indicated concentrations of PD, SP, and SB for 30 min before treatment with 10 μM KMU-191 for 24 h. (F) Cells were treated with KMU-191 for 24 h in the presence or absence of N-acetylcysteine or glutathione ethyl ester. sub-G1 population and protein expression were measured by flow cytometry (A) and Western blotting analysis (C,D), respectively. Cleavage of PARP is indicated with an arrowhead. The expression level of β-actin was used as a control of protein loading. Values in the graph (A-F) indicate the mean ± SD of three independent experiments. * P < 0.05 compared to the respective control. HCT116 p53 (+/+), p53 wild-type HCT116 cells; HCT116 p53 (-/-), p53 deficient HCT116 cells; NAC, N-acetylcysteine; GEE, glutathione ethyl ester.

To determine the functional importance of c-FLIP (L) and Mcl-1 (L) in KMU-191-mediated apoptosis, we used human clear cell renal cell carcinoma Caki cells engineered to overexpress c-FLIP (L) and Mcl-1 (L). As shown in Figure 4A, c-FLIP (L) overexpression partially inhibited KMU-191-mediated apoptosis. Moreover, the Mcl-1 (L) overexpression completely suppressed KMU-191-mediated apoptosis (Figure 4B). Next, to investigate the functional role of p53 in KMU-191-mediated apoptosis in cancer cells, we used Caki cells transfected with siRNA targeting p53 mRNA and HCT116 cells (p53 wild-type and p53 deficient). Immunoblot analysis demonstrated that transfection with p53 siRNA suppressed p53 expression in Caki cells compared to that in cells transfected with control GFP siRNA (Figure 4C). Remarkably, KMU-191-mediated apoptosis and PARP cleavage were inhibited in Caki cells transfected with p53 siRNA compared to those in control GFP siRNA-transfected Caki cells (Figure 4C). Moreover, apoptosis and PARP cleavage were inhibited in KMU-191-treated p53 deficient HCT116 cells, but not in p53 wild-type HCT116 cells (Figure 4D).

To explore MAPK signaling, which plays an important role in cell proliferation, survival, and differentiation 21, 22, during KMU-191-induced apoptosis, we used specific MAPK inhibitors. However, none of the specific MAPK inhibitors (PD, MEK inhibitor; SP, JNK inhibitor; SB, p38 MAPK inhibitor) attenuated KMU-191-induced apoptosis in Caki cells (Figure 4E). Reactive oxygen species (ROS), byproducts of normal oxygen metabolism, play an important role in apoptosis under both physiologic and pathologic processes 23. Therefore, we investigated the role of ROS in the apoptosis of Caki cells induced by KMU-191. As shown in Figure 4F, KMU-191-induced apoptosis was not suppressed by pretreatment with NAC and GEE. These results demonstrated that KMU-191-induced apoptosis is not associated with either MAPK signaling pathways or ROS.

To evaluate whether KMU-191 have cytotoxic effect in normal cells, we used TCMK-1 and MC. KMU-191 induced apoptosis of TCMA-1 and MC cells in a dose-dependent manner (Figure 4G and 4H). In addition, KMU-191 induced PARP cleavage, up-regulation of p53 and p21 in TCMK-1 cells (Figure 4G). These results suggested that the cytotoxicity of KMU-191 is induced by similar mechanisms in cancer cells and normal cells.

### The effect of KMU-191 on cardiotoxicity assessment

**Figure 5:**
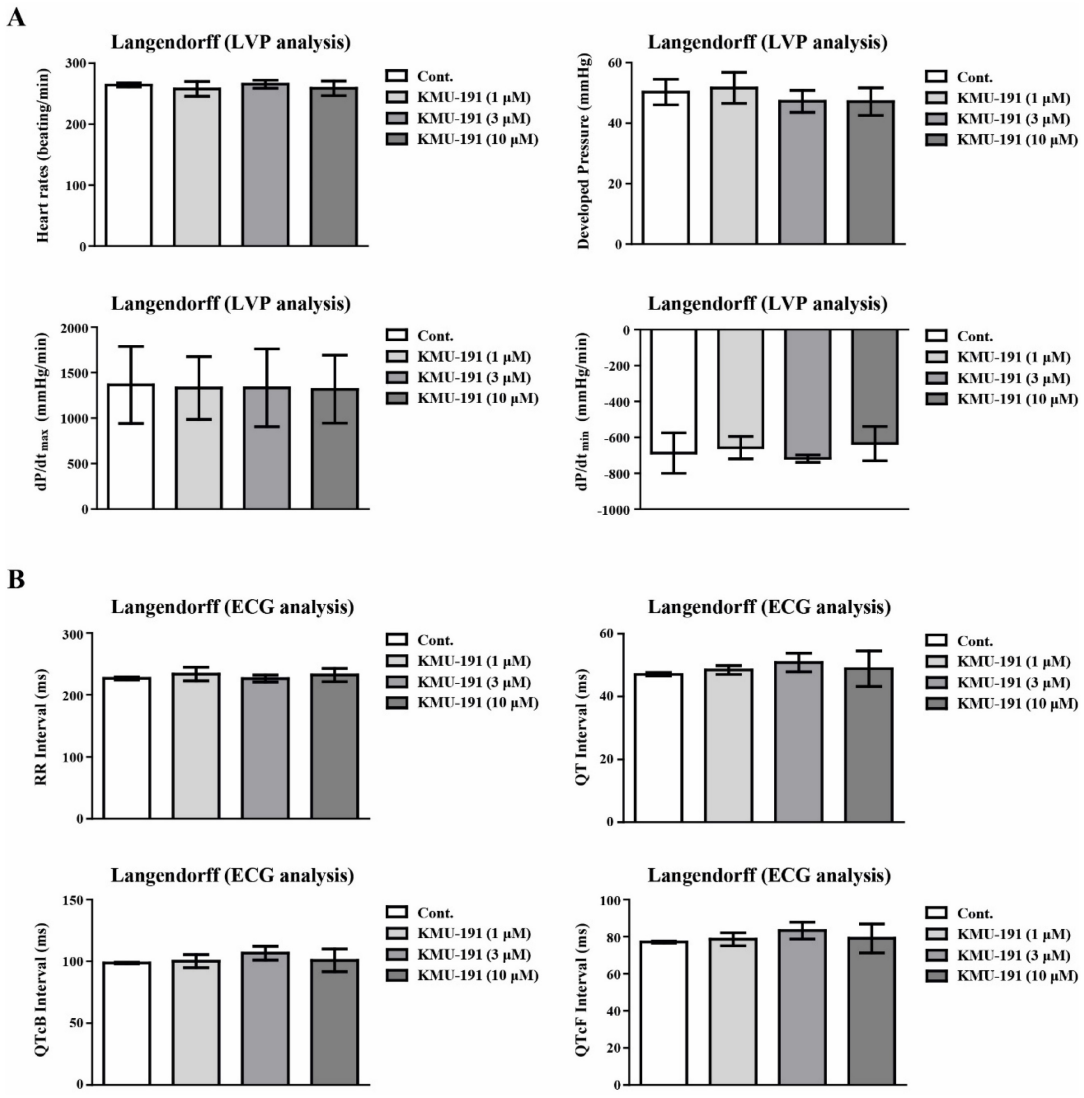
** Assessment of electrophysiological safety of KMU-191.** The isolated perfused Sprague Dawley rats heart was respectively conditioned with 0.1% dimethyl sulfoxide as control and KMU-191 (1, 3, and 10 µM). (A) Effect of KMU-191 on left ventricle pressure analysis. (B) Effect of KMU-191 on electrocardiogram analysis. Values in the graph (A,B) indicate the mean ± SD of three independent experiments. Cont., control.

Many anticancer drugs cause cardiotoxicity as a side effect 24, 25. We assessed cardiotoxicity in a dose-dependent manner (1 - 10 µM) to explore whether KMU-191 causes cardiotoxicity. To assess cardiotoxicity, we used the Langendorff heart system for LVP and ECG analyses. We analyzed cardiotoxicity factors such as heart rate, developed pressure, maximal rate of contraction and relaxation (dP/dt_max_ and dP/dt_min_), ECG waveform RR Interval, QT Interval, QT correction Bazett (QTcB) Interval, and QT correction Fridericia (QTcF) Interval. As shown in Figure 5A, KMU-191 did not affect any of the hemodynamic parameters in the LVP analysis. In addition, the results of the ECG analysis showed that KMU-191 dose not induce cardiotoxicity in a dose-dependent manner (Figure 5B). Thus far, these results suggested that KMU-191 has the potential to induce apoptosis of cancer cells without causing cardiotoxicity.

## Discussion

Human genes encode over 500 protein kinases 26, and dysregulated protein kinases are associated with carcinogenesis 27. Among the various anti-cancer drugs targeting dysregulated kinases 28, aminoindazole-based small molecules have been investigated as a strategy for cancer treatment 1-4. We previously reported the synthesis of a novel aminoindazole-based compound, 2-(4-Ethoxyphenyl)-*N*-(5-((2-fluoro-4-(morpholine-4-carbonyl)phenyl)amino)-1*H*-indazol-3-yl)acetamide, and its anti-cancer effects on various human cancer cells 1. In this study, we found that KMU-191 effectively induces caspase-dependent apoptosis and regulates apoptosis-related proteins in human renal cell carcinoma Caki cells.

Although targeted anti-cancer therapy has attracted attention as an effective therapeutic strategy 29, there is an unmet need to provide more efficacious cancer treatments due to intratumoral heterogeneity 30. For this reason, the development of multi-target drugs capable of regulating various targets involved in cancer pathophysiology has received considerable attention 31, 32. Dysregulation of anti-apoptotic Bcl-2 family proteins, such as Bcl-xL and Mcl-1, is associated with the survival of cancer cells and resistance to anti-cancer drugs 33, 34. Moreover, c-FLIP overexpression frequently occurs in various types of human cancers and is associated with inhibition of cell death receptor-mediated apoptosis 35, 36. As shown in Figure 2C, 2D and 3B-D, KMU-191 induced down-regulation of c-FLIP (L) at the transcriptional level, and Mcl-1 (L) and Bcl-xL at the post-transcriptional level. Additionally, Mcl-1 (L) overexpression completely suppressed KMU-191-induced apoptosis. In contrast, c-FLIP (L) overexpression partially suppressed the KMU-191-induced apoptosis. Taken together, these results suggest that Mcl-1 (L) down-regulation is involved in KMU-191-induced apoptosis and that KMU-191 could overcome the c-FLIP (L)-mediated survival effect in Caki cells.

Nuclear or cytoplasmic accumulation of the tumor suppressor p53 is well known as an important mechanism of DNA damage-induced apoptosis 37. In this study, KMU-191 induced p53 up-regulation in a dose- and time-dependent manner (Figure 2C and 2D). Remarkably, p53 siRNA blocked KMU-191-induced apoptosis in Caki cells (Figure 4C). Moreover, KMU-191 induced apoptosis in cancer cells of various status of p53 gene (Figure 2C, 2E, and 2F). Interestingly, KMU-191 induced G2/M arrest in p53 deficient HCT116 cells (Figure 2G). These results suggest that p53 up-regulation plays an important role in KMU-191-induced apoptosis and KMU-191 has anti-proliferative effect of cancer cells regardless of p53 status.

Among the apoptosis pathways in cancer cells, ROS generation and the MAPK pathways are well known as important regulators in the establishment of cancer therapeutic strategies 38, 39. Therefore, we tested specific inhibitors to evaluate the involvement of ROS generation and MAPK pathways in KMU-191-mediated apoptosis. However, none of the specific inhibitors reduced KMU-191-induced apoptosis in Caki cells (Figure 4E and 4F).

Cardiovascular toxicity is a major factor limiting the development and clinical application of this class of drugs, such as kinase inhibitors 40. Cardiovascular toxicity and safety assessments such as LVP and ECG are required to evaluate the possibility of inducing arrhythmia during drug development 41. Therefore, we determined the cardiovascular toxicities of KMU-191 using ECG and LVP analyses using the Langendorff test. The Langendorff heart preparation method is one of the screening *ex vivo* models for evaluating the proarrhythmic toxicity of test substance on the heart by excluding external influences from humoral and neural factors 42. In addition, according to the ICH S7B guideline, which describes a non-clinical testing strategy for assessing the proarrhythmic risk of drugs, the Langendorff heart preparation method is known as a good method to evaluate the proarrhythmic risk of drugs 43. As shown in Figure 5, KMU-191 had little few potential risks associated with cardiotoxicity, suggesting that KMU-191 has the potential to be developed as an anti-cancer drug.

Our study had some limitations that should be investigated more closely. First, given that KMU-191 is a kinase inhibitor, the mechanism of kinase inhibition of KMU-191 and its relevance to the up- and down-regulation of proteins have not been fully explored. Additionally, the sensitivity to KMU-191 among the cells used differed, so it is necessary to compare which genetic backgrounds exhibit differences in sensitivity to KMU-191. Although KMU-191 had anti-cancer effect in Caki, it had also cytotoxicity in normal cell lines such as TCMK-1 and MC. Finally, we did not validate the anti-cancer efficacy of KMU-191 in vivo. Therefore, further studies are needed to elucidate the deeper mechanisms of anti-cancer effect of KMU-191, and compound optimization studies to reduce cytotoxicity in normal cells are necessary.

Despite the limitations of this study, we found that KMU-191 induced apoptosis in various cancer cells by activating caspase-3, down-regulating Bcl-xL, Mcl-1 (L), and c-FLIP (L), and up-regulating p53. Moreover, we demonstrated the electrophysiological safety of KMU-191 *ex vivo*. Collectively, these results suggest that KMU-191 is a clinically useful and attractive multi-target agent against cancer.

## Figures and Tables

**Table 1:** Primer Sequences

Gene name	Sequences^*^
Bcl-xL sense	5'-GGTCGCATTGTGGCCTTT-3'
Bcl-xL antisense	5'-TCCTTGTCTACGCTTTCCACG-3'
c-FLIP (L) sense	5'-TGCTGAAGTCATCCATCAGG-3'
c-FLIP (L) antisense	5'-ATTCCTAGGGGC TTGCTCT-3'
Mcl-1 (L) sense	5'-GCGACTGGCAAAGCTTGGCCTCAA-3'
Mcl-1 (L) antisense	5'-CAACTCTAGAAACTGGTTTTGGTG-3'
β-actin sense	5'-AATCTGGCACCACACCTTCTA-3'
β-actin antisense	5'-ATAGCACAGCCTGGATAGCAA-3'

^*^Primer Sequences of apoptosis related-genes used in qPCR

## Abbreviations

c-FLIP: cellular FADD-like IL-1β-converting enzyme inhibitory protein
Mcl-1: myeloid cell leukemia 1
Bcl-2: B cell lymphoma-2
Bcl-xL: B-cell lymphoma-extra large
TdP: Torsade de pointes
ECG: electrocardiography
LVP: left ventricle pressure
ATCC: American Type Culture Collection
FBS: fetal bovine serum
DMEM: Dulbecco's modified Eagle's medium
GEE: glutathione ethyl ester
PARP: poly (ADP-ribose) polymerase
Z-VAD-FMK: Benzyloxycarbony-Val-Ala-Asp-fluoromethyl ketone
PD: PD-98059
SP: SP600125
SB: SB-203580
NAC: N-acetylcysteine
SRB: Sulforhodamine B
PBS: Phosphate-buffered saline
qPCR: Quantitative real-time PCR
siRNA: Small interfering RNA
ROS: reactive oxygen species
QTcB: QT correction Bazett
QTcF: QT correction Fridericia

## References

[1] Lee J, Choi H, Kim KH, Jeong S, Park JW, Baek CS (2008). Synthesis and biological evaluation of 3,5-diaminoindazoles as cyclin-dependent kinase inhibitors. Bioorganic & medicinal chemistry letters.

[2] Dai Y, Hartandi K, Ji Z, Ahmed AA, Albert DH, Bauch JL (2007). Discovery of N-(4-(3-amino-1H-indazol-4-yl)phenyl)-N'-(2-fluoro-5-methylphenyl)urea (ABT-869), a 3-aminoindazole-based orally active multitargeted receptor tyrosine kinase inhibitor. Journal of medicinal chemistry.

[3] Medina JR, Blackledge CW, Heerding DA, Campobasso N, Ward P, Briand J (2010). Aminoindazole PDK1 Inhibitors: A Case Study in Fragment-Based Drug Discovery. ACS Med Chem Lett.

[4] Maggio B, Raimondi MV, Raffa D, Plescia F, Cascioferro S, Plescia S (2011). Synthesis of substituted 3-amino-N-phenyl-1H-indazole-1-carboxamides endowed with antiproliferative activity. Eur J Med Chem.

[5] Lee J, Kim J, Hong VS, Park JW (2014). Synthesis and anti-proliferative activity evaluation of N3-acyl-N5-aryl-3,5-diaminoindazole analogues as anti-head and neck cancer agent. Daru.

[6] Domingos PM, Steller H (2007). Pathways regulating apoptosis during patterning and development. Curr Opin Genet Dev.

[7] Elmore S (2007). Apoptosis: a review of programmed cell death. Toxicol Pathol.

[8] Morioka S, Maueroder C, Ravichandran KS (2019). Living on the Edge: Efferocytosis at the Interface of Homeostasis and Pathology. Immunity.

[9] Tower J (2015). Programmed cell death in aging. Ageing Res Rev.

[10] Kashyap D, Garg VK, Goel N (2021). Intrinsic and extrinsic pathways of apoptosis: Role in cancer development and prognosis. Adv Protein Chem Struct Biol.

[11] Green DR, Llambi F (2015). Cell Death Signaling. Cold Spring Harb Perspect Biol.

[12] Yoshida K, Miki Y (2010). The cell death machinery governed by the p53 tumor suppressor in response to DNA damage. Cancer science.

[13] Ferdinandy P, Baczko I, Bencsik P, Giricz Z, Gorbe A, Pacher P (2019). Definition of hidden drug cardiotoxicity: paradigm change in cardiac safety testing and its clinical implications. Eur Heart J.

[14] Ben-David J, Zipes DP (1993). Torsades de pointes and proarrhythmia. Lancet.

[15] Norton K, Iacono G, Vezina M (2009). Assessment of the pharmacological effects of inotropic drugs on left ventricular pressure and contractility: an evaluation of the QA interval as an indirect indicator of cardiac inotropism. J Pharmacol Toxicol Methods.

[16] Redfern WS, Carlsson L, Davis AS, Lynch WG, MacKenzie I, Palethorpe S (2003). Relationships between preclinical cardiac electrophysiology, clinical QT interval prolongation and torsade de pointes for a broad range of drugs: evidence for a provisional safety margin in drug development. Cardiovasc Res.

[17] Forero-Cortés PA, Haydl AM (2019). The 25th anniversary of the Buchwald-Hartwig amination: development, applications, and outlook. Organic Process Research & Development.

[18] Olivier M, Eeles R, Hollstein M, Khan MA, Harris CC, Hainaut P (2002). The IARC TP53 database: new online mutation analysis and recommendations to users. Hum Mutat.

[19] Park JG, Yang HK, Kim WH, Chung JK, Kang MS, Lee JH (1997). Establishment and characterization of human gastric carcinoma cell lines. International journal of cancer.

[20] Schmittgen TD, Livak KJ (2008). Analyzing real-time PCR data by the comparative C(T) method. Nature protocols.

[21] Kim EK, Choi EJ (2010). Pathological roles of MAPK signaling pathways in human diseases. Biochimica et biophysica acta.

[22] Low HB, Zhang Y (2016). Regulatory Roles of MAPK Phosphatases in Cancer. Immune Netw.

[23] Simon HU, Haj-Yehia A, Levi-Schaffer F (2000). Role of reactive oxygen species (ROS) in apoptosis induction. Apoptosis: an international journal on programmed cell death.

[24] McGowan JV, Chung R, Maulik A, Piotrowska I, Walker JM, Yellon DM (2017). Anthracycline Chemotherapy and Cardiotoxicity. Cardiovasc Drugs Ther.

[25] Renu K, V GA, P BT, Arunachalam S (2018). Molecular mechanism of doxorubicin-induced cardiomyopathy - An update. Eur J Pharmacol.

[26] Fabbro D, Cowan-Jacob SW, Moebitz H (2015). Ten things you should know about protein kinases: IUPHAR Review 14. Br J Pharmacol.

[27] Kannaiyan R, Mahadevan D (2018). A comprehensive review of protein kinase inhibitors for cancer therapy. Expert Rev Anticancer Ther.

[28] Dancey J, Sausville EA (2003). Issues and progress with protein kinase inhibitors for cancer treatment. Nat Rev Drug Discov.

[29] Sawyers C (2004). Targeted cancer therapy. Nature.

[30] Gerlinger M, Rowan AJ, Horswell S, Larkin J, Endesfelder D, Gronroos E (2012). Intratumor heterogeneity and branched evolution revealed by multiregion sequencing. N Engl J Med.

[31] Ling Y, Liu J, Qian J, Meng C, Guo J, Gao W (2020). Recent Advances in Multi-target Drugs Targeting Protein Kinases and Histone Deacetylases in Cancer Therapy. Curr Med Chem.

[32] Sunil D, Kamath PR (2017). Multi-Target Directed Indole Based Hybrid Molecules in Cancer Therapy: An Up-To-Date Evidence-Based Review. Curr Top Med Chem.

[33] Walensky LD (2006). BCL-2 in the crosshairs: tipping the balance of life and death. Cell Death Differ.

[34] Yamaguchi R, Lartigue L, Perkins G (2019). Targeting Mcl-1 and other Bcl-2 family member proteins in cancer therapy. Pharmacology & therapeutics.

[35] Bagnoli M, Canevari S, Mezzanzanica D (2010). Cellular FLICE-inhibitory protein (c-FLIP) signalling: a key regulator of receptor-mediated apoptosis in physiologic context and in cancer. The international journal of biochemistry & cell biology.

[36] Safa AR (2012). c-FLIP, a master anti-apoptotic regulator. Exp Oncol.

[37] Green DR, Kroemer G (2009). Cytoplasmic functions of the tumour suppressor p53. Nature.

[38] Dhillon AS, Hagan S, Rath O, Kolch W (2007). MAP kinase signalling pathways in cancer. Oncogene.

[39] Ivanova D, Bakalova R, Lazarova D, Gadjeva V, Zhelev Z (2013). The impact of reactive oxygen species on anticancer therapeutic strategies. Adv Clin Exp Med.

[40] Lamore SD, Kohnken RA, Peters MF, Kolaja KL (2020). Cardiovascular Toxicity Induced by Kinase Inhibitors: Mechanisms and Preclinical Approaches. Chem Res Toxicol.

[41] Oren O, Neilan TG, Fradley MG, Bhatt DL (2021). Cardiovascular Safety Assessment in Cancer Drug Development. J Am Heart Assoc.

[42] Watanabe M, Okada T (2018). Langendorff Perfusion Method as an Ex Vivo Model to Evaluate Heart Function in Rats. Methods Mol Biol.

[43] Guideline IHT (2005). The non-clinical evaluation of the potential for delayed ventricular repolarization (Qt Interval Prolongation) by human pharmaceuticals. S7B (http://www ich org/products/guidelines/safety/article/safety-guidelines html).

